# Changes in Physicochemical, Free Radical Activity, Total Phenolic and Sensory Properties of Orange (*Citrus sinensis* L.) Juice Fortified with Different Oleaster (*Elaeagnus angustifolia* L.) Extracts

**DOI:** 10.3390/molecules27051530

**Published:** 2022-02-24

**Authors:** Maryam Sarvarian, Afshin Jafarpour, Chinaza Godswill Awuchi, Ademiku O. Adeleye, Charles Odilichukwu R. Okpala

**Affiliations:** 1 Department of Food Science & Technology, Savadkooh Branch, Islamic Azad University, Savadkooh 47418-39959, Iran; 2 Department of Food Science & Technology, Garmsar Branch, Islamic Azad University, Garmsar 35816-31167, Iran; afjapo@gmail.com; 3 Department of Biochemistry, Kampala International University, Bushenyi P.O. Box 20000, Uganda; awuchichinaza@gmail.com; 4 Faith Heroic Generation, No. 36 Temidire Street, Akure 340251, Ondo State, Nigeria; adeleyedsegun@gmail.com; 5 Department of Functional Foods Product Development, Wrocław University of Environmental and Life Sciences, 51-630 Wrocław, Poland

**Keywords:** *Citrus sinensis*, *Elaeagnus angustifolia*, natural antioxidant, extraction solvent, physicochemical properties

## Abstract

In Iran and other parts of Western Asia, the oleaster (*Elaeagnus angustifolia* L.) fruit is processed in the dried powdery form, and in recent times, increasingly applied/sprinkled in fruit juices such as those made from oranges (*Citrus sinensis* L.). To our best knowledge, the effectiveness of oleaster fruit extract in fortifying the orange juice has not yet been reported and the knowledge of this will greatly benefit the consumers, particularly those around the Western Asia region. This current work, therefore, investigated the changes in physicochemical, free radical activity, total phenolic compounds, and sensory properties of orange juice fortified with different oleaster fruit extracts. The orange juice mix formulation comprised different concentrations (5, 10, 15, 20, and 25%) of oleaster (alcoholic, aqueous, and hydro-alcoholic) extracts. The control comprised orange concentrate (4% *w*/*v*), sugar (8.5% *w*/*v*), and citric acid (0.1% *w*/*v*) brought to the desirable volume with water. As the free radical activity depicted the antioxidant properties, the physicochemical aspects of this work involved the determinations of Brix, density, ash, pH, total acidity, sucrose, and total sugar, whereas the sensory aspects involved the determinations of color and taste. Whilst the aqueous oleaster 20 and 25% extracts produced notable physicochemical differences in the orange juice mix, both free radical activity, and phenolic compounds significantly increased (*p* < 0.05) after 30 days despite resembling (*p* > 0.05) those of control at day 1. More so, the increases in aqueous, alcoholic, and hydro-alcoholic oleaster extracts would decrease (*p* < 0.05) the sensory color and taste of the orange juice mix in this study.

## 1. Introduction

Globally, the food given to the human body increasingly receives a great deal of attention [1,2,3]. On one hand, consuming food rich in antioxidants can reduce the development of chronic diseases and oxidative stress and its associated risks. On the other hand, the high levels of the reactive oxygen species (ROS) would cause the imbalance of antioxidants as well as prooxidants, which tends more to be in favor of the latter (i.e., prooxidants) [1]. That is why, as many people worldwide increasingly pursue a healthier lifestyle, the functionality of fruit juices is increasingly being studied [2]. More so, fruit juices comprise bioactive health-promoting and disease-reducing components vital to human metabolism and wellbeing [4]. Despite being processed from fresh harvests and consumed directly, the natural fruit juices remain highly prone to deterioration, which makes their fresh and stable production very challenging [4,5]. The transformation of a given fruit into juice form primarily focuses to extend its shelf time. Such transformation could also allow the incorporation of additives/spices, which act as fortificants [4,5,6,7]. Natural juice additives can include the use of active peptides, anti-browning agents, antimicrobials, essential oils, and their components, as well polyphenols [4]. Besides the antimicrobial and antioxidant properties associated with natural compounds in fruits, the addition of natural/herbal extracts is believed not to adversely affect the consumer acceptance (of fruit juices) [6,7].

With respect to orange juice, the sweet fruit type *Citrus sinensis* (L.) remains largely dominant, despite being dependent on the species as well as varieties [8]. Typically, when the orange fruit is processed to a juice form, it is classified as a fruit drink [9]. Globally, orange juice remains very popular with an annual production of roughly 63 million tons [10,11]. Moreover, orange juice provides an important dietary source of bioactive compounds, such as phenolic compounds, carotenoids, ascorbic acid (AA), and vitamin C, that contribute to its antioxidant properties [12]. Of increasing interest among some researchers is the oleaster (*Elaeagnus angustifolia* L.) fruit of the Elaeagnaceae family and obtained from a shrub/tree [13,14], reported in Iranian folk medicine for its anti-inflammatory and analgesic functions, and distributed widely from the Himalayas and Europe to the northern regions of Asia [15]. The edible fruits, consumed fresh or dried, are a rich source of vitamins such as tocopherol, vitamin C, B1, and α-carotene, as well as minerals (potassium, sodium, and phosphorous) [3]. Oleaster fruit, commonly called wild olive, silverberry, Russian olive, or oleaster, is a species of Elaeagnus native to Iran (commonly called Senjed), and Western and Central Asia [13,14,16]. Specifically, the high antioxidant properties of oleaster have been demonstrated by the significant amounts of flavonoid, terpenoid, cytosterol, carvacrol, glucose, fructose, phenolic, and caffeic acid compounds [13,14,17,18,19]. Additionally, it is worth highlighting that the antioxidant activity of plant extracts can be determined in such diverse ways as: ABTS (2,2-azinobis (3-ethyl-benzothiazoline-6-sulphonate)), DPPH free radical (scavenging) (2,2-diphenyl-1-picrylhydrazyl), ESR (electron spin resonance), FRAP (ferric reducing antioxidant power), and ORAC (oxygen radical absorbance capacity) [20].

Bioactive compounds in fruits are usually derived through extraction processes. Whilst the extraction methods used to identify the bioactive components in fruits are varied, the cheaper and more affordable approaches are usually sought after [21,22]. In fact, the extraction process primarily aims not only to achieve the very best amounts of the target compounds, but also to actualize most of the biological activity of these extracts [22]. Considering the phenolic compound recovery from plant material as an example, solvent extraction appears the most commonplace [23]. The choice of extraction technique together with the extraction solvent, besides both affecting the resultant biological activity and yield [22], directly influences the selectivity, and consequently, the final extract based on chemical composition and functional properties [24]. Moreover, to experimentally characterize and quantify the properties of solvents is achievable via the three solvatochromic Kamlet–Taft parameters, namely: (1) hydrogen-bond donating ability (acidity, α); (2) hydrogen-bond accepting ability (basicity, β); and (3) polarizability/polarity (π *) [25]. Additionally, many solvents are being employed to extract bioactive compounds from various plant materials such as fruits. Examples of solvents include methanol, ethanol, acetone, and water [22]. For instance, water appears an interesting viable solvent in the extraction process given the changes that happen in its chemical and physical properties at varying temperatures [21]. Indeed, some researchers have demonstrated ethanol/water solvents as more effective in extracting phenolic compounds compared to water, whereas the ethanol extracts showed higher antioxidant activity compared to aqueous extracts [26]. Further, some researchers have recommended ethanol–water mixtures for the preparation of plant extracts given their acceptability for human consumption [23]. Moreover, hydro-alcoholic mixtures are believed to serve as good extraction candidates. This is because they are considered rather selective and believed to possess a wide range of polarities regarding the compounds that can be extracted [24].

Several efforts, which appear even more in recent years, have been aimed to better the understanding that underpins natural compounds found in food products, particularly regarding their applicability, functionality, and properties [27]. As plant materials comprise a variety of bioactive compounds with differing solubility properties, the appropriateness of solvent for extraction would be dependent on the specificity (of plant material), as well as compounds aimed for [22]. Moreover, there is an increase in consumer demand for high-quality orange juice with natural taste, favorable textures, minimal additives [28], and increased beneficial properties for health/wellbeing [7,29]. More so, and particularly across Iran and spread to other parts of the Western Asia regions, the oleaster fruit is being processed into the dried powdery form and applied/sprinkled onto milk with the aim to fortify it [14]. Additionally, nowadays, it is increasingly being applied/sprinkled into fruit juices such as orange. To our best knowledge, the effectiveness of oleaster fruit extract in fortifying the orange juice is not yet reported, and the knowledge of this will greatly benefit the consumers, particularly those around the Western Asia region. Therefore, this current work investigated the changes in physicochemical, free radical activity, total phenolic compounds, and sensory properties of orange (*Citrus sinensis* L.) juice fortified with different oleaster (*E. angustifolia* L.) extracts. For emphasis, together with the free radical activity and total phenolic compounds, the physicochemical aspects determined the Brix, density, ash content, total acidity, total sugar, pH, and sucrose, whereas the sensory aspects determined the color and taste.

## 2. Results and Discussion

### 2.1. Compositional Differences of Oleaster Extracts

The compositional differences of oleaster (*E. angustifolia*) extracts are given in Table 1. For emphasis, the oleaster extracts have been based on three different solvents, that is, aqueous, alcoholic, and hydro-alcoholic (extracts). Results showed the aqueous extract obtained significantly more (*p* < 0.05) ash, Brix, density, total sugar, and sucrose of oleaster compared to the others. However, the alcoholic extract, despite having significantly lower (*p* < 0.05) ash, density, total acidity, total acidity, sugar, sucrose, and total sugar contents, had pH levels that were of significantly higher (*p* < 0.05) values compared to the other treatments. From these results of Table 1, we opine that the aqueous extraction method projects somewhat higher promise compared to the others. Probably, the increased polarity of the solvent, that is, from water, water–ethanol, to ethanol, could contribute to the enhancement of the physicochemical properties of the oleaster extract of this current study. Moreover, the hard nature of the cell wall of the oleaster might play a key role towards the permeability of water, which would be key in making effective the extraction of (bioactive) compounds [30]. Besides factors such as extraction conditions, as well as solvent concentration/type to significantly affect the extraction efficiency, the applied solvent will also influence the composition of plant extract, and consequently its biological activity [23].

### 2.2. Changes in Physicochemical Properties

**Figure 1:**
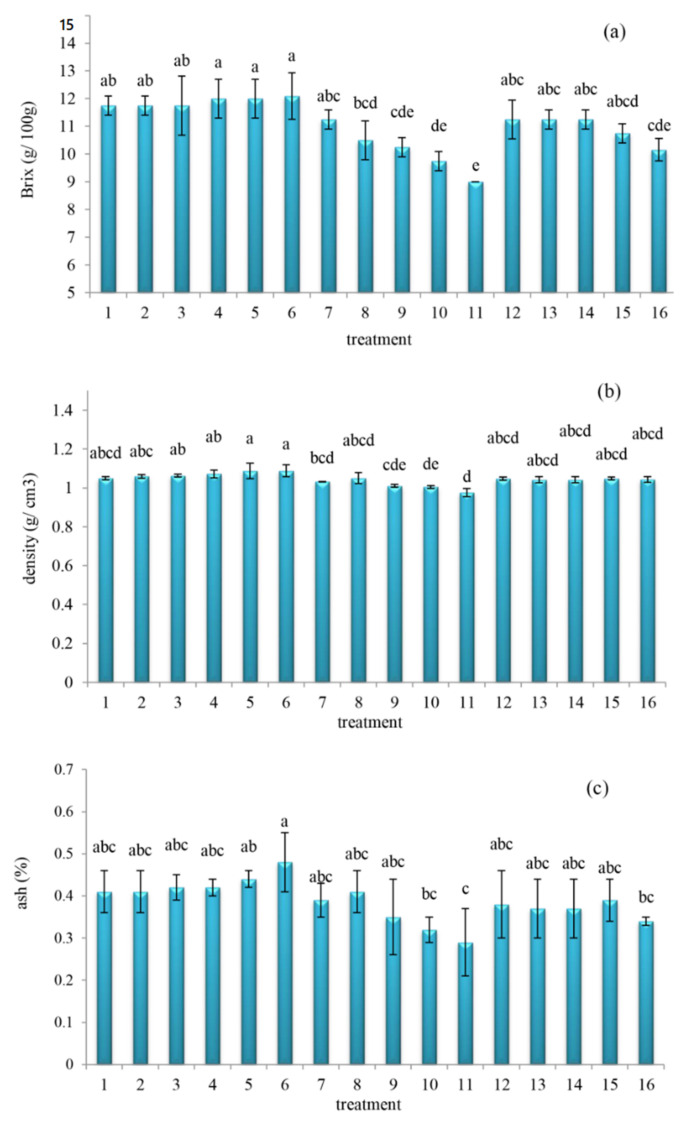
Changes in Brix (**a**), density (**b**), and ash (**c**) values of the orange juice subject to different oleaster extracts ((1) control, (2) aqueous extract 5%, (3) aqueous extract 10%, (4) aqueous extract 15%, (5) aqueous extract 20%, (6) aqueous extract 25%, (7) alcoholic extract 5%, (8) alcoholic extract 10%, (9) alcoholic extract 15%, (10) alcoholic extract 20%, (11) alcoholic extract 25%, (12) hydro-alcoholic extract 5%, (13) hydro-alcoholic extract 10%, (14) hydro-alcoholic extract 15%, (15) hydro-alcoholic extract 20%, (16) hydro-alcoholic extract 25%). Different letters (a–e) indicate significant changes occurred at *p* < 0.05.

The changes in soluble solids (Brix), density, and ash values of orange juice subject to different oleaster extracts are shown in Figure 1. Further, the ANOVA readings showed statistically significant variations in the soluble solids (Brix) (Figure 1a) (*p* = 0.001, F-change = 5.285), density (Figure 1b) (*p* = 0.032, F-change = 2.632), and ash (Figure 1c) (*p* = 0.023, F-change = 1.349) across different treatments of the orange juice mix, with respective ranges between 9 and 12 g/100 g, 1.088–1.01 g/cm^3^, and 0.29–0.48%. Additionally, the Brix, density, and ash values of the control orange juice sample appeared at roughly 11.75 g/100 g, 1.049 g/cm^3^, and 0.41%, respectively. Comparing Figure 1a,c both Brix and ash trended somewhat similarly across the treatments. Whereas the alcoholic extract 25% obtained the least Brix, density, and ash values, the aqueous extract 25% obtained the highest Brix, density, and ash values. Moreover, the increased ash values, obtained by the aqueous extract 25%, confirm the oleaster fruit as the promising mineral source. Additionally, Boudraa et al. [31] reported fruits of *E. angustifolia* L. enriched with vitamins such as carotene, thiamine B1, tocopherol, vitamin C, together with minerals such as calcium (Ca), iron (Fe), magnesium (Mg), manganese (Mn), and potassium (K). Additionally, Hashemi et al. [7] showed that the addition of *Moringa oleifera* leaf extract to orange juice increased the amount of dry matter in the drink. These workers considered such increases to indicate the beverages were enriched with the micronutrients added by the (*M. oleifera* leaf) extracts.

**Figure 2:**
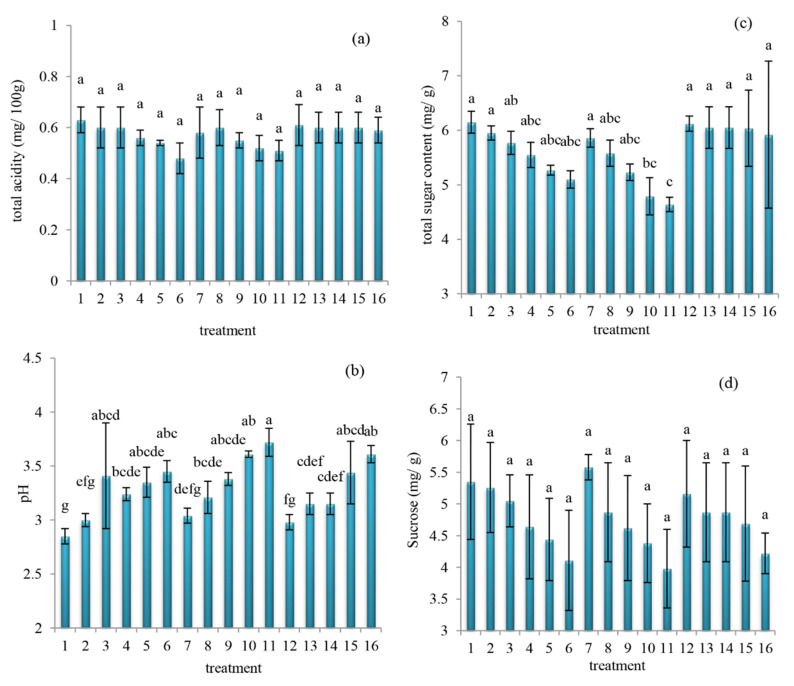
Changes in total acidity (**a**), pH (**b**), total sugar content (**c**), and sucrose (**d**) values of orange juice subject to different oleaster extracts ((1) control, (2) aqueous extract 5%, (3) aqueous extract 10%, (4) aqueous extract 15%, (5) aqueous extract 20%, (6) aqueous extract 25%, (7) alcoholic extract 5%, (8) alcoholic extract 10%, (9) alcoholic extract 15%, (10) alcoholic extract 20%, (11) alcoholic extract 25%, (12) hydro-alcoholic extract 5%, (13) hydro-alcoholic extract 10%, (14) hydro-alcoholic extract 15%, (15) hydro-alcoholic extract 20%, (16) hydro-alcoholic extract 25%). Different letters (a–g) indicate significant changes occurred at *p* < 0.05.

Notably, the critical factor affecting the juice spoilage, among others, includes the pH. Additionally, the microbial spoilage in fruit juices has been represented by cloud loss, CO_2_ production, off-flavor development, as well as sensorial changes, which would bring about a loss in the product value [32,33]. The changes in total acidity, pH, total sugar content, and sucrose values of orange juice subject to different oleaster extracts are shown in Figure 2. Further, the ANOVA readings showed statistically significant variations in the pH (Figure 2b) (*p* = 0.003, F-change = 4.411) and sugar content (Figure 2c) (*p* = 0.041, F-change = 2.470) across different treatments of the orange juice mix, but not so at total acidity (Figure 2a) (*p* = 0.644, F-change = 0.823) and sucrose (Figure 2d) (*p* = 0.653, F-change = 0.813). Despite this, the total acidity (Figure 2a), pH (Figure 2b), total sugar content (Figure 2c), and sucrose (Figure 2d) values obtained ranges of 0.48–0.63 mg/100 g, 2.85–3.72, 4.64–6.15 mg/g, and 3.98–5.98 mg/g, respectively. Probably, the addition of oleaster extracts somewhat decreased the total acidity of the orange juice mix, but not significantly (*p* > 0.05). Additionally, the acid-binding properties (of the oleaster extract, in this context) might have decreased the total acidity [29] of the orange juice mix. Moreover, the addition of various oleaster extracts significantly increased (*p* < 0.05) the pH values at different rates. Specifically, these pH increases were found more steeply at the alcoholic, compared to the aqueous/hydro-alcoholic extracted ones. However, we found both total sugar content and sucrose to significantly decrease (*p* < 0.05), except at those of the hydro-alcoholic that did not change (*p* > 0.05) despite the increasing pH that took place because of the addition of the various oleaster extracts. Similarly, Hashemi et al. [7] reported the addition of *M. oleifera* leaf extract increased the pH and decreased the total acidity of orange juice.

Additionally, the increase in pH values may be due to the citric acid (acidity) decrease, which has been reported elsewhere [33,34]. In the current work, both total acidity (~0.63 mg/100 g) and total sugar content (~6.15 mg/g), respectively, peaked at the control and hydro-alcoholic oleaster extract 25% (refer to Figure 2a,c). The obtained total acidity range (0.48–0.63 mg/100 g) herein fell above that previously detected at the initial stage of orange juice (0.45 mg/100 g) reported by Bull et al. [11], which might be attributed to reasons such as differences in either season, geographic area, and/or plant variety. Moreover, Gloria et al. [35] understood that fruit extracts could bring about peak and low (total) acidity, respectively, at the beginning and during/end of a season. More so, there appears to be a similar trend when comparing total acidity (Figure 2a), total sugar content (Figure 2c), and sucrose (Figure 2d) of this current study. In addition, Akhtar et al. [36] understood that the sucrose content in fruit juice could be affected by the acidity.

### 2.3. Changes in DPPH Free Radical Activity and Total Phenolic Compounds

**Figure 3:**
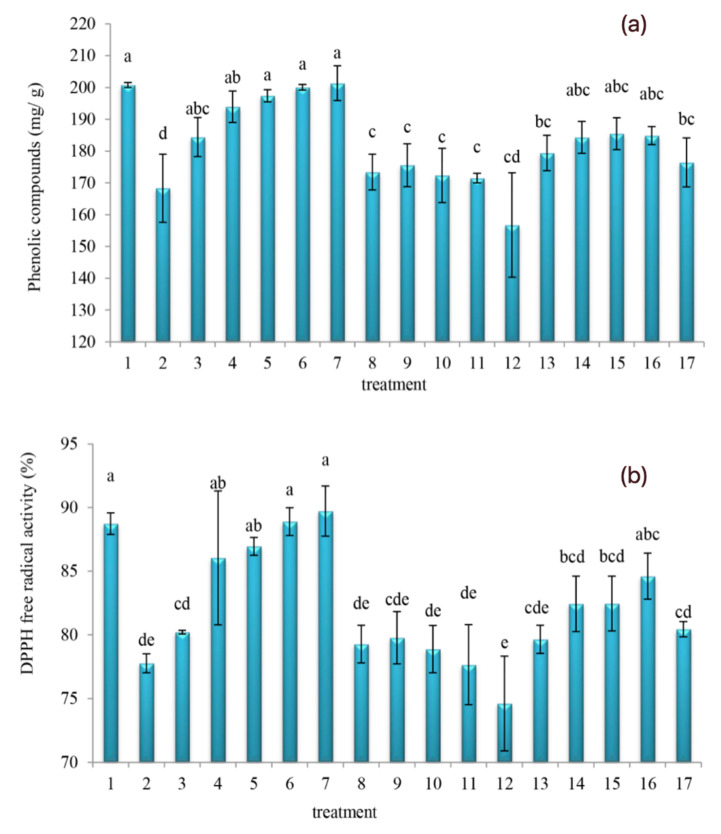
Changes in total phenolic compounds (**a**) and DPPH free radical activity (**b**) values of orange juice subject to different oleaster extracts ((1) control, (2) aqueous extract 5%, (3) aqueous extract 10%, (4) aqueous extract 15%, (5) aqueous extract 20%, (6) aqueous extract 25%, (7) alcoholic extract 5%, (8) alcoholic extract 10%, (9) alcoholic extract 15%, (10) alcoholic extract 20%, (11) alcoholic extract 25%, (12) hydro-alcoholic extract 5%, (13) hydro-alcoholic extract 10%, (14) hydro-alcoholic extract 15%, (15) hydro-alcoholic extract 20%, (16) hydro-alcoholic extract 25%). Different letters (a–e) indicate significant changes occurred at *p* < 0.05.

Different authors have recommended the DPPH assay as an accurate/easy method to measure the antioxidant activity of orange juice, as well as those made from other fruit products [33,37,38]. For emphasis, the oleaster extracts have been added in this study given our aim to produce a beneficial orange juice that would become enriched with both phenolic compounds and antioxidant properties. Therefore, as a means of comparing the control and extraction methods, it was needful to determine the DPPH free radical activity and total phenolic compounds at day 30. The changes in total phenolic compounds and DPPH free radical activity values of orange juice subject to different oleaster extracts are shown in Figure 3. Further, the ANOVA readings showed statistically significant variations in the total phenolic compounds (Figure 3a) (*p* = 0.000, F-change = 6.060) and DPPH free radical activity (Figure 3b) (*p* = 0.000, F-change = 8.339) across different treatments of the orange juice mix. Moreover, the DPPH free radical activity and total phenolic compounds of control significantly decreased (*p* < 0.05) from ~88.72 to 77.77%, and from 200.75 to 168.32 mg GAE/g, respectively. The results were in agreement with data reported by Romeo et al. [33], where the antioxidant activity in orange juice decreased during storage time. Between the aqueous oleaster extract 25% and alcoholic extract 5%, together with the control, more resemblances (*p* > 0.05) in DPPH free radical activity and total phenolic compounds were found. Nonetheless, the addition of aqueous oleaster extract would significantly increase (*p* < 0.05) the DPPH free radical activity and total phenolic compounds. Different from the aqueous extract, the addition of alcoholic and hydro-alcoholic oleaster extracts would produce significantly lower (*p* < 0.05) DPPH free radical activity and total phenolic compounds despite their somewhat initial stable values.

Given the increased presence of hydroxyl groups within the reaction medium [39,40], the increases in the total phenolic compounds would corroborate with the ability of different (oleaster) extracts to enhance the free radical activity. Comparing total phenolic compounds (Figure 3a) and DPPH free radical activity (Figure 3b), we can observe a resembling trend. This (observed resembling trend) might suggest the antioxidant power corroborated the quantities of total phenolic compounds in the oleaster-fortified orange juice mix of this current study. Indeed, the total phenolic compounds in the plants with high antioxidant power should be more extractable [41,42]. Previous studies of oleaster extracts have shown it to possess significant quantities of antioxidants and promising photochemical compounds, such as phenols [17,18,19,43]. The oleaster fruits and leaves have been shown to produce flavonoid- and polyphenol-containing extracts. The methanol extracts of the oleaster genotypes, through the assays of the free radical activity as well as total flavonoid/phenolic contents, would demonstrate antioxidant and antiradical activities. Compared to the flesh and peels, there could be the situation where the fruit seeds obtain promising antioxidant activity and higher phenolic contents [3]. Therefore, it should not be surprising that the oleaster hydro-alcoholic extract 5% provided the least DPPH free radical activity and total phenolic compounds to the orange juice mix (refer to Figure 3).

The peaks of both total phenolic compounds and DPPH free radical activity appears to demonstrate the efficacy of the aqueous extract method of this study, as shown in Figure 3. Specifically, it is promising that the aqueous 20 and 25% as well as alcoholic 5% oleaster extract provided increased DPPH free radical activity and total phenolic compounds to the orange juice mix. Hashemi et al. [7] revealed that adding *M. oleifera* leaf extract to orange juice increased its phenolic compounds and antioxidant properties. Moreover, other researchers [40,41,42,43,44] have demonstrated the amounts of extracted phenolic compounds from different plants tend to portray the polarity of the solvents used, together with their associated solubility. Essentially, how such (polarity–solubility) situations would interact with other constituents in the plant tissues may well, in our opinion, justify why solvents in this current study might have delivered such differences in the total phenolic compounds. Moreover, the solubility of the extraction solvent could be influencing the degree of both hydrophilic and hydrophobic properties of phytochemical compounds. Therefore, the solvent polarity would be key if the extraction efficiency of these phytochemical compounds is to be assured [42,44]. For emphasis in this current work, the polarity of the water solvent played its role especially in the extraction of useful amounts of antioxidants and phenolic compounds [43].

### 2.4. Changes in Sensory Attributes

**Figure 4:**
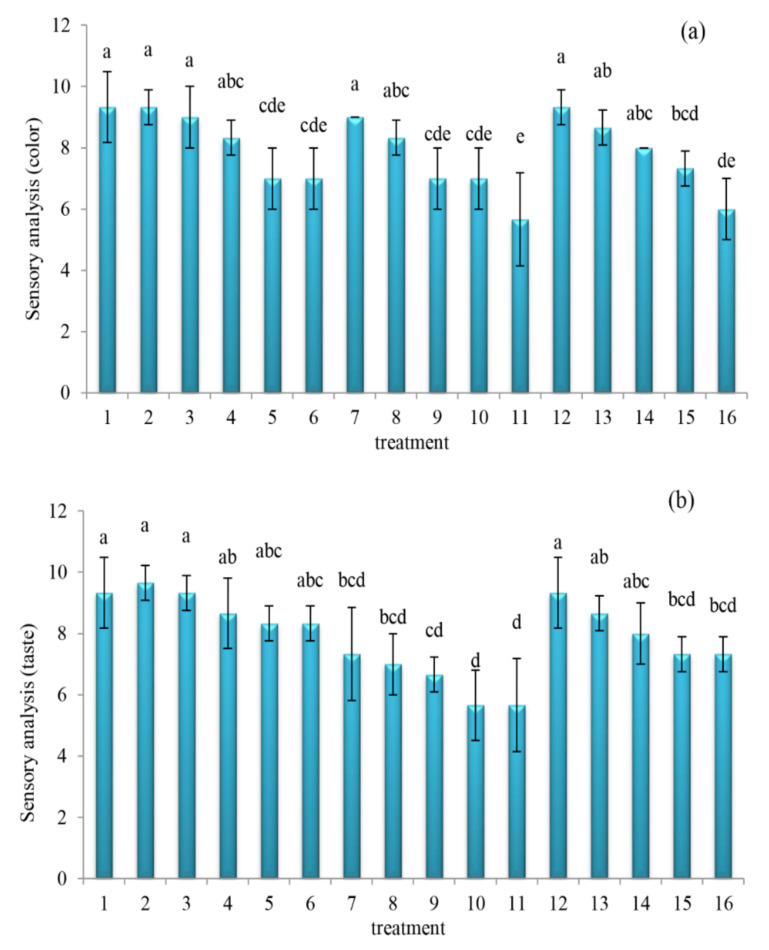
Changes in sensory color (**a**) and taste (**b**) values of orange juice subject to different oleaster extracts ((1) control, (2) aqueous extract 5%, (3) aqueous extract 10%, (4) aqueous extract 15%, (5) aqueous extract 20%, (6) aqueous extract 25%, (7) alcoholic extract 5%, (8) alcoholic extract 10%, (9) alcoholic extract 15%, (10) alcoholic extract 20%, (11) alcoholic extract 25%, (12) hydro-alcoholic extract 5%, (13) hydro-alcoholic extract 10%, (14) hydro-alcoholic extract 15%, (15) hydro-alcoholic extract 20%, (16) hydro-alcoholic extract 25%). Different letters (a–e) indicate significant changes occurred at *p* < 0.05.

The changes in sensory color and taste values of orange juice subject to different treatments are shown in Figure 4. Further, ANOVA readings showed statistically significant variations in the sensory color (Figure 4a) (*p* = 0.000, F-change = 5.956) and taste (Figure 4b) (*p* = 0.000, F-change = 11.894) across different treatments of the orange juice mix. Moreover, the addition of the oleaster extracts as a fortifier significantly affected the sensory color and taste of the orange juice samples. The sensory color (Figure 4a) and taste (Figure 4b) was peak at control, and subsequently decreased with increasing concentration of oleaster. Sensory color values of control resembled (*p* > 0.05) those of aqueous extract 5 and 10%, alcoholic extract 5%, as well as hydro-alcoholic extract 5%. Sensory color resemblances were so for taste (*p* > 0.05), with the exception of alcoholic extract 5% that was significantly lower (*p* < 0.05). Regarding juice taste, the highest sensory score was related to aqueous oleaster extract 20 and 25%. Furthermore, the lowest sensory score was observed in all extracts of alcoholic extract 25%. The oleaster aqueous extracts 20 and 25% obtained a color sensory score of about 7, and an after-taste sensory score of about 8.33. Feasibly, it can be said that the aqueous extract 25% treatment provided a rather flavored taste.

Having a further look at Figure 3 and Figure 4, we observe there appears an interesting connection when both DPPH free radical scavenging and total phenolic compounds are compared with the sensory color and taste attributes. Specifically, on one hand, the peak values of the DPPH free radical activity and total phenolic compounds at aqueous oleaster extract 20 and 25% of orange juice mix (refer to Figure 3), despite resembling that of its control, tend to corroborate the somewhat lower sensory color and taste when compared to its control (refer to Figure 4). On the other hand, the least values of DPPH free radical activity and total phenolic compounds of hydro-alcoholic oleaster extract 5%, despite being significantly (*p* < 0.05) lower than that of its control (refer to Figure 3), tends to corroborate a different sensory color and taste that resembled (*p* > 0.05) that of its control (refer to Figure 4). Fan et al. [45] understood that the use of descriptive analysis combined with chemical analysis can help identify the chemical drivers of sensory attributes. These authors considered that the combined analyses would bring about discoveries regarding the relationships between sensory intensities and flavor-active compounds. Moreover, by comparing Figure 1, Figure 2 and Figure 4 of this current work, it is not that straightforward to decipher the degree of association/corroboration between the physicochemical and sensory attributes. We opine that the higher quantities of aqueous, alcoholic, and hydro-alcoholic oleaster extracts might have contributed to significantly (*p* < 0.05) decrease the sensory color and taste values of the orange juice mix in this study. Probably, this impact of lower sensory scores may well corroborate the higher pH (refer to Figure 2b), as well as the lower total sugar content and sucrose values (refer to Figure 2c,d, respectively) of the orange juice mix in this study.

## 3. Materials and Methods

### 3.1. Schematic Overview of the Experimental Program

**Figure 5:**
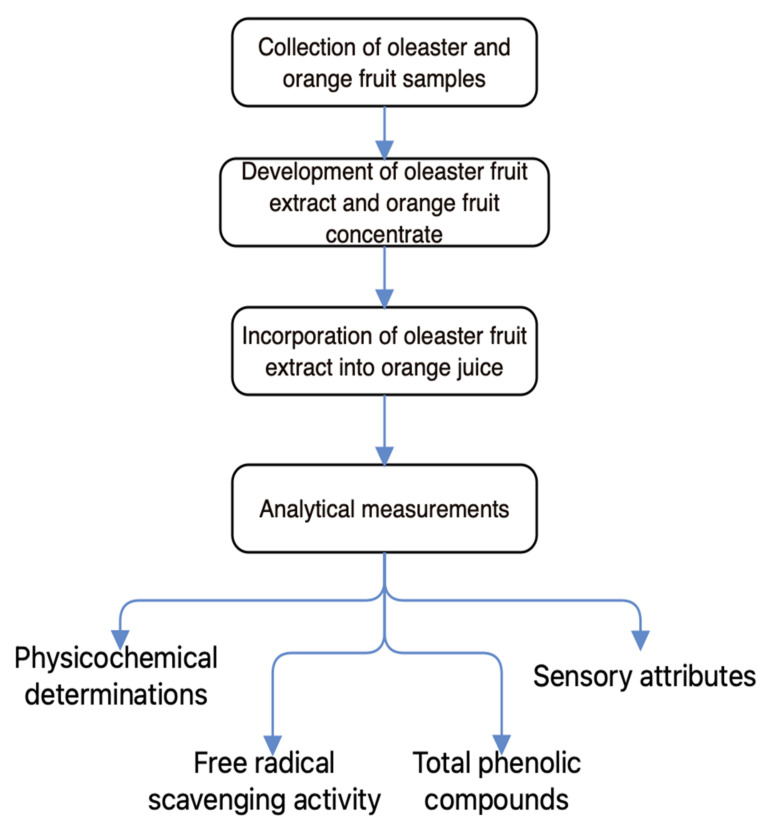
A schematic overview of experimental program, indicating the major stages of this current work, from the collection of oleaster and orange fruit samples, the development of oleaster fruit extract and orange fruit concentrate, the incorporation of oleaster fruit extract into orange juice, to the analytical measurements.

A schematic overview of the experimental program is displayed in Figure 5, which reveals the key stages of this current work, from the collection of oleaster and orange fruit samples, the development of oleaster fruit extract and orange fruit concentrate, the incorporation of oleaster fruit extract into orange juice, to the analytical measurements. We aimed, through this current work, to produce a beneficial orange juice mix enriched with antioxidant and phenolic compounds, which would effectively compete with the ordinary orange juice, particularly in terms of customer friendliness and quality. The process to make the oleaster extract followed standard laboratory procedures. Additionally, the orange juice mix was prepared under aseptic conditions.

### 3.2. Chemical and Reagents

All chemicals and reagents for this current work were of analytical grade standard. They included acetone, iodine, starch, Parafilm, Fehling solution, Erlenmeyer titrate, sodium hydroxide procured from Merck (Merck KGaA, Darmstadt, Germany). Others included formaldehyde solution, Folin–Ciocalteu reagent, calcium hardness (Calcio) reagent procured from Fisher (Fisher Scientific GmbH, Vienna, Austria), as well as sodium carbonate, and methanolic 2,2-diphenyl-1-picrylhydrazyl (DPPH) solution procured from Sigma-Aldrich (Sigma-Aldrich, St. Louis, MO, USA).

### 3.3. Identification of Oleaster and Orange Fruits

**Figure 6:**
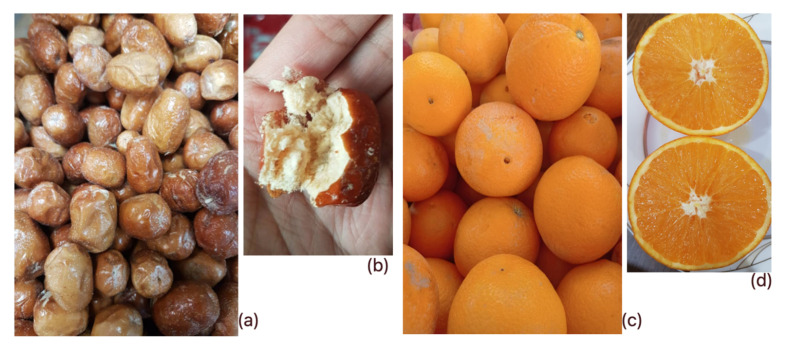
A pictorial representation of the samples of oleaster fruits (**a**) and a view of one when opened (**b**), as well as the samples of orange fruits (**c**) and views of a sectioned slice (**d**).

The oleaster fruit (*E. angustifolia* L.) was collected from the north of the Damghan region, Alborz mountain belt, Northern Iran. Identification of the studied oleaster species was performed using the opinions of the experts at the Cultivation and Development Department, Institute of Medicinal Plants, Iran. Additionally, the orange fruit type (*C. sinensis* L.) used in this study is the typical sweet type, which are being cultivated by the Ramsar citrus concentrate factory, Ramsar, Iran. For emphasis, Figure 6 shows a pictorial representation of the samples of oleaster fruits (Figure 6a) and a view of one when opened (Figure 6b), as well as the samples of orange fruits (Figure 6c) and views of a sectioned slice (Figure 6d).

### 3.4. Development of the Oleaster Fruit Extract and Orange Fruit Concentrate

The preparation of the oleaster fruit powder mimicked those of artisans found in Iran and other Western Asia regions. The oleaster fruit samples have been dried in a vacuum oven at 50 °C for 48 h and completely powdered by a shredder and kept at 25 °C until the experiment. The oleaster fruit extract was made with three different solvents such as water (aqueous extraction), ethanol (alcoholic extraction), and water (50%)–ethanol (50%) (hydro-alcoholic extraction), as prescribed by the Institute of Standards and Industrial Research of Iran [46]. This required about 150 g of oleaster powder obtained from whole fruit, which were first milled with the kernel, and thereafter, added to 1000 mL of boiling components (three different solvents were added separately) at room temperature (1:10 *w*/*v*), and thereafter, kept in the dark for about 48 h. The extraction was repeated three times and the solvent was evaporated in a vacuum until the dryness of extracts and dried extracts have been stored in a glass container in the dark at 4 °C temperature until required [47]. Additionally, the orange concentrate was purchased from the sales branch of Pars Caravan Company, Tehran, Iran. The orange concentrate was prepared following the procedures already authenticated by the Institute of Standards and Industrial Research of Iran [46], as is used at the Ramsar citrus concentrate factory, Ramsar, Iran. The orange concentrate, after its procurement, was freeze stored at −18 °C until required for the experiment.

### 3.5. Incorporation of Oleaster Fruit Extracts into Orange Juice Mix

To prepare the control treatment sample, the (sweet) orange concentrate (4% *w*/*v*), sugar (8.5% *w*/*v*), and citric acid (0.1% *w*/*v*) were mixed, and thereafter, brought to the desirable volume with water. Importantly, the dense nature of this orange concentrate aligns with a specified Brix, which enables it to mix with the desired extract when arranged in ordinary water [47]. For preparation of other treatments, different concentrations of aqueous, alcoholic, and hydro-alcoholic extracts with respective proportions of 5, 10, 15, 20, and 25% were supplemented to the orange juice formulation. Pectin gum (0.1%) (Danisco A/S, 8220 Brabrand, Denmark) helped to create the uniformity and reduce the two-phase degradation of the oleaster orange mix. Subsequently, the mix samples were then placed in the bain-marie, where the water subject to ample heating had stabilized at 98 ± 2 °C, and this was held for 15 min, to pasteurize the samples. The bain-marie was equipped with a temperature-controlled thermometer to ensure adequate monitoring and stability (of temperature) and this lasted for about 15 min. Thereafter, the oleaster orange mix samples were placed on the pasteurized lid briefly (~30 s), and subsequently in ice-cold water (~2 min) for rapid cooling, which was aimed at minimizing any damage to the samples. Subsequently, the mix samples were subjected to dark-refrigeration at 4 ± 2 °C for a 30-day storage period specific to this current study. Additionally, the orange juice mix was prepared under aseptic conditions.

### 3.6. Analytical Methods

#### 3.6.1. Physicochemical Measurements

##### Determination of Brix and Density

The Brix was determined using the method described by Martín-Diana et al. [28] with slight modifications. This involved a standardization process of the refractometer device with the help of distilled water until the Brix was set to the zero mark. Thereafter, a few drops of samples were placed on the sensitive surface of the refractometer lens, and readings were collected. The results were expressed in terms of one gram of sucrose in 100 g of (sucrose) solution. The density of sample was determined using the method described by He et al. [48], using a 50 mm pycnometer with a thermometer set at 20 °C, with the scale accuracy of 0.001 g. The results were expressed in terms of mg/100 g.

##### Determination of Ash Content

The ash content was determined using the AOAC [49] method with slight modifications. All porcelain capsules (~50 cm^3^) used had been placed in the high temperature muffle furnace to achieve complete dryness, then cooled in the desiccator, weighed, and recorded. About 25 mL of juice in a pre-weighed crucible was placed on a 100 °C baking tray to drain the water. Then, a few drops of pure olive oil were added and allowed to slowly burn on the flame until it did not smoke. Thereafter, the capsule was placed in the high temperature muffle furnace at 525 °C until the white ash was obtained. After cooling, the sample was weighed before and after to determine the concentration of ash present, which has been expressed in g/100 g (wet weight) of the sample as prescribed by the Institute of Standards and Industrial Research of Iran [46].

##### Determination of Total Acidity

Due to the color of the sample and the lack of timely detection of discoloration in the measurement of acidity by titration, the total acidity was measured by the potentiometric method [50]. Firstly, the pH meter was calibrated. The beaker was then placed on a magnetic stirrer, with the electrode of a pH meter gently beside it. Then, the pH meter and the magnetic stirrer were switched on, and the burette valve was gently opened until a drop of 0.1 N of sodium hydroxide was added. As the normal sodium hydroxide pH of the sample reached 8.1, the volume (of sodium hydroxide) consumed was recorded. The total acidity of the sample was calculated using the equation provided by the Iranian National Standard No. 2685 [46]:

(1) $$Acidity=\frac{v\times 0.0064\times 100}{m}$$

where *v* = consumption volume of 0.1 N sodium hydroxide in milliliters; *m* = sample weight in g; and total acidity presented in terms of citric acid in g per hundred g.

##### Determination of Total Sugar Contents

The total sugar content of samples was determined using the Iranian National Standard No. 2685 [46] with slight modifications. Approximately 25 mL of distilled water was added to a 100 mL graduated balloon previously filled with 25 mL of filtered solution. Then, while spinning the balloon, 6 to 10 mL of hydrochloric acid was added, the water bath was heated to 60–70 °C, and adjusted to a temperature of approximately 70 °C for 3 min, and balloon placed in another bath of 20 °C and stirred constantly for 7 min until balloon contents cooled to 35 °C. At that point, the contents had neutralized with the phthalate, in the presence of phenol, which had increased to volume with water. About 5 mL of Fehling A, and 5 mL of Fehling B in Erlenmeyer titrate 100 mL, consistent as above-mentioned, after which the total sugar contents per 100 g of sample was determined, with the help of the equation below:

(2) $$Total\;sugar\;content=\frac{100\times 100\times 100\times Fehling\;factor}{Consumption\;volume\;of\;solution\;\left(mL\right)\times 25\times 25}$$

##### Determination of pH

The pH was determined using the method described by Martín-Diana et al. [28] with slight modifications. Approximately 20 mL of each orange juice sample was poured into 100 mL Erlenmeyer (at room temperature) and then the pH of the samples was measured with a digital pH meter calibrated to standards at pH 4 and 7.

##### Determination of Sucrose

The amount of sucrose expressed in terms of one gram of content per 100 g of sample was calculated according to the method prescribed by the Iranian National Standard (No. 2685) [46]:

Sucrose = (N − n) 0.95(3)

where ‘N’ is G percent of total sugar; ‘n’ is G of regenerating sugar; 0.95 is the ratio of the molecular weights of sucrose and those of glucose and fructose.

#### 3.6.2. Determination of DPPH Free Radical Activity

The DPPH free radical activity test was determined using the method described by Su and Silva [51] with slight modifications. About 0.3 mL of each extract was added to 3.7 mL of methanolic DPPH solution (6 × 10^5^ mol/L) and the resulting mixture was stirred vigorously. After 30 min of darkening at room temperature, the absorbance of the sample was recorded at 517 nm with the help of a UV-Visible spectrophotometer (Evolution™ 201/220, Thermo Scientific, Rochester, NY, USA). All of these steps were performed on tert-Butylhydroquinone (TBHQ) as a standard antioxidant and samples (methanolic DPPH solution prepared in addition to the relevant solvents). The DPPH free radical activity (*DPPH*) was determined using the equation below:

(4) $$DPPH\;free\;radical\;activity\left(\%\right)=\frac{\left(Absorbance\;of\;blank–Absorbance\;of\;sample\right)}{\left(Absorbance\;of\;blank\right)}\times 100$$

#### 3.6.3. Determination of Total Phenolic Compounds

The amount of total phenolic compounds of samples was determined using the spectroscopic method with Folin–Ciocalteu reagent as described by Esmaeilzadeh et al. [41], with slight modifications. Then, 0.5 mL of the above-prepared extract was mixed with 2.5 mL of a 10-fold diluted Folin–Ciocalteu reagent, together with 2 mL of 7.5% sodium carbonate solution. Subsequently, and at room temperature, the samples were kept for 30 min followed by the absorbance reading conducted spectrometrically (UV-Visible spectrophotometer, Evolution™ 201/220, Thermo Scientific, Rochester, NY, USA) to determine the total phenolic compounds. Additionally, the number of phenolic compounds has been determined in control at day 0, and day 30, and similarly at day 30 on all other treatments. The results have been expressed in terms of the milliequivalents of gallic acid per gram of extract [42].

#### 3.6.4. Determination of Sensory Attributes

The sensory evaluation was performed on the samples using the method described by Meilgaard et al. [52] with slight modifications. The sensory panelists comprised 10 (ten) trained students/staff of the Department of Food Science and Technology, Islamic Azad University at the Savadkooh Branch. Prior to participation, the panelists underwent sensory training to make them experienced with the (sensory) evaluation criteria applicable to typical orange juice. Prior to their participation, verbal consent was taken. To ensure their privacy, neither name nor gender was indicated. The sensory panelists were individually provided with the evaluation forms. Each sample was coded, where one code represented each treatment. Additionally, each panelist had ample space with no contact with the next, so that one opinion did not influence the other. Each panelist evaluated the samples for two sensory attributes, that is, color and taste. All sensory attributes were recorded on 10-point hedonic scale, with 1 (least liked) to 10 (most liked). Consistent with Çakmakçı et al. [53], each sensory panelist cleansed their palates between samples with clean warm water, which ensured that the evaluation of the previous sample would not influence that of the new sample.

### 3.7. Statistical Analysis

All emergent data were subject to one-way analysis of variance (ANOVA) and reported as mean ± standard deviation (SD) of three measurements. The mean comparisons were conducted using Duncan’s multiple range test (DMRT). The level of probability was defined at *p* < 0.05, and when obtainable, the F-change values were also reported. Statistical Package for the Social Sciences (SPSS) package (version 18, SPSS Inc, Chicago, IL, USA) was used to run the data.

## 4. Conclusions

The changes in physicochemical, free radical activity, total phenolic and sensory properties of orange (*C. sinensis* L.) juice fortified with different oleaster (*E. angustifolia* L.) extracts were investigated in this study. It was shown that, whereas the alcoholic oleaster extract 25% obtained the least Brix, density, and ash values, the aqueous oleaster extract 25% obtained the highest values. Increases in oleaster extracts somewhat decreased acidity, but increased the pH values, both at different rates, and more steeply at those of the alcoholic compared to those of the aqueous/hydro-alcoholic. Additionally, the aqueous oleaster extract significantly increased with the phenolic compounds and free radical activity values. As the sensory color and taste values both peaked at the control, they would decrease subsequently with increased concentration of oleaster.

To reiterate, the oleaster has been added because the target in this study was to produce a beneficial orange juice mix that contained competitive and promising phenolic compounds/antioxidant properties. Therefore, we considered it needful to measure the total phenolic compounds and DPPH free radical activity at day 30, which has allowed effective comparisons between the extraction methods and control. Given the results of the total phenolic compounds and DPPH free radical activity of this current study, future study should also be directed on shelf-life evaluation of orange juice mix fortified with different oleaster extracts under different storage conditions. Another direction of future studies could be to conduct high-performance liquid chromatography (HPLC) (or high-performance thin-layer chromatography (HPTLC)) profiles of oleaster (*E. angustifolia* L.) extracts, in the view to provide additional information regarding their bioactive components and help substantiate their efficacy. All the emergent data of such above-mentioned studies would help to validate the effectiveness of the aqueous, alcoholic, and hydro-alcoholic oleaster extraction methods used in this study, and supplement existing literature.

## Figures and Tables

**Table 1:** Compositional differences of oleaster (*E. angustifolia*) extract.

Parameters	Aqueous Extract	Alcoholic Extract	Hydro-Alcoholic Extract
Brix (g/100 g)	8.25 ± 1.43 ^a^	2.35 ± 0.07 ^c^	5.75 ± 0.09 ^b^
Density (g/cm^3^)	1.20 ± 0.12 ^a^	0.83 ± 0.03 ^c^	1.04 ± 0.05 ^b^
Ash (%)	0.47 ± 0.03 ^a^	0.012 ± 0.003 ^c^	0.13 ± 0.02 ^b^
Total acidity (mg/100 g)	0.34 ± 0.03 ^b^	0.17 ± 0.02 ^c^	0.54 ± 0.13 ^a^
pH	5.25 ± 0.35 ^b^	6.95 ± 0.23 ^a^	5.85 ± 0.21 ^a,b^
Total sugar content (mg/g)	1.88 ± 0.16 ^a^	0.23 ± 0.04 ^c^	0.95 ± 0.11 ^b^
Sucrose (mg/g)	0.38 ± 0.16 ^a^	0.11 ± 0.01 ^c^	0.25 ± 0.06 ^b^

Different small letters (^a,b,c^) in the same row represent significant difference (*p* < 0.05).

## Data Availability

Not applicable.

## Sample Availability

The reported oleaster extracts can be made available, upon reasonable request, by authors M.S. and A.J.
